# Diversity and Functional Roles of Root-Associated Endophytic Fungi in Two Dominant Pioneer Trees Reclaimed from a Metal Mine Slag Heap in Southwest China

**DOI:** 10.3390/microorganisms12102067

**Published:** 2024-10-15

**Authors:** Bo Bi, Yuqing Xiao, Xiaonan Xu, Qianqian Chen, Haiyan Li, Zhiwei Zhao, Tao Li

**Affiliations:** 1 State Key Laboratory for Conservation and Utilization of Bio-Resources in Yunnan, Yunnan University, Kunming 650091, China; bibo@stu.ynu.edu.cn (B.B.); 18960879821@163.com (Y.X.); xiaonanxu2021@163.com (X.X.); chenqianqian477@163.com (Q.C.); 2 Medical School, Kunming University of Science and Technology, Kunming 650504, China; lhyxrn@163.com

**Keywords:** phytomanagement, dark septate endophyte (DSE), mine tailing soil, revegetation, *Populus yunnanensis*

## Abstract

The utilization of fast-growing, economically valuable woody plants with strong stress resistance, such as poplar and willow, to revegetate severely metal-contaminated mine tailings not only offers a productive and profitable use of abandoned polluted soil resources but also facilitates the phytoremediation of these polluted soils. This study examines the diversity and functional roles of endophytic fungi naturally colonizing the roots of an artificially established *Populus yunnanensis* forest and the naturally reclaimed pioneer species *Coriaria sinica* on an abandoned tailing dam in southwest China. Culture-independent analyses revealed that the root systems of both plant species were abundantly colonized by arbuscular mycorrhizal fungi and endophytic fungi, forming rich and diverse endophytic fungal communities predominantly represented by the genera *Ilyonectria*, *Tetracladium*, *Auricularia*, and unclassified members of Helotiales. However, the composition of root endophytic fungal communities differed significantly between the two plant species. Using a culture-dependent approach, a total of 192 culturable endophytic fungal strains were isolated from the roots. The dominant genera included *Cadophora*, *Cladosporium*, *Cyphellophora*, and *Paraphoma,* most of which were previously identified as dark septate endophytes (DSE). Six representative DSE strains were selected for further study, and significant cadmium tolerance and various plant growth-promoting traits were observed, including the solubilization of insoluble inorganic and organic phosphorus, indole-3-acetic acid (IAA) production, and siderophore synthesis. In greenhouse experiments, inoculating two DSE strains mitigated the inhibitory effects of metal-polluted tailing soil on the growth of *P. yunnanensis*. This was achieved by reducing heavy metal uptake in roots and limiting metal translocation to the above-ground tissues, thereby promoting plant growth and adaptability. Our findings suggest that as plants reclaim metal-polluted tailings, root-associated endophytic fungal communities also undergo natural succession, playing a critical role in enhancing the host plant’s tolerance to stress. Therefore, these restored root-associated fungi, particularly DSE, are essential functional components of the root systems in plants used for tailing reclamation.

## 1. Introduction

Plant roots serve as rich reservoirs for various microorganisms, some of which form symbiotic relationships with their host plants, such as arbuscular mycorrhizal fungi and root-associated non-mycorrhizal fungal endophytes [1,2]. Among these, dark septate endophytic fungi (DSEs) have gained significant attention [3]. DSEs are characterized by their melanized septate hyphae and microsclerotia, which can grow both inside and between the epidermal and cortical cells of plant roots [4]. DSEs are widely distributed across different plant groups, including bryophytes, pteridophytes, and angiosperms, and are found in various habitats, from arctic and alpine regions to the Antarctic and temperate zones [5,6,7]. Numerous studies have shown that DSEs are often found in extreme environments, such as polar regions, highly saline areas, drought-prone zones, and ecosystems contaminated with heavy metals [5,8]. For example, in a study of 23 cushion plants and associated species in the high Chilean Andes, Casanova-Katny et al. [9] found that more plant species were colonized by DSEs than by arbuscular mycorrhizal fungi (AMF), with DSE colonization increasing with altitude. Similarly, typical DSE structures were observed in the roots of 31 non-mycorrhizal plant species in heavy metal tailing soils in Yunnan Province, southwest China [10]. Netherway et al. [11] also proposed that DSEs may play a more pivotal role in shaping tree-associated microbiomes than mycorrhizal colonization or abiotic factors in widespread broad-leaved trees across a European latitudinal gradient, with DSE colonization explaining significant variation in fungal and bacterial communities in soil and roots, as shown by permutational multivariate analysis of variance (PERMANOVA).

DSEs are well adapted to harsh environments and can enhance the stress tolerance of host plants in various ways. For example, Li et al. [12] found that inoculating the DSE strain isolated from the drought-tolerant plant *Gymnocarpos przewalskii* enhanced the growth of the non-xerophyte *Ammopiptanthus mongolicus* under drought conditions. Similarly, inoculating the DSE strain *Phialocephala bamuru* A024 improved resistance to damping-off disease in host *Pinus sylvestris* var. *mongolica* seedlings and altered the bacterial community in the rhizosphere, thereby improving soil nutrient status and increasing host biomass [13].

Recent studies confirm the significant and diverse functions of DSEs. These fungi secrete various enzymes that help plants absorb essential nutrients like carbon, nitrogen, and phosphorus. They also enhance plant photosynthetic efficiency, promote growth, and increase seedling survival rates [7,14]. DSE strains can synthesize auxins, which directly regulate plant rhizome growth or alter the host plant’s endogenous hormones [15,16], thereby improving stress resistance, particularly against heavy metals [17,18]. For example, Hou et al. [19] reported that DSE inoculation enhanced root growth and nutrient uptake in non-host plants under cadmium (Cd) stress, by altering Cd concentrations in the soil. Additionally, DSEs can synergistically interact with other beneficial microbes, like ectomycorrhizal fungi, to improve root architecture, growth, and Cd tolerance, thus enhancing the survival of host *Pinus tabulaeformis* in metal-contaminated environments [20]. Interestingly, while AMF colonization decreases under heavy metal stress, DSE colonization tends to remain stable or even increase in polluted soils [11,21], suggesting that DSEs have strong potential for use in plant-microbial restoration efforts [18,22].

Plants growing in metal tailing areas, such as obligate or facultative metallophyte species, develop high resistance to heavy metals through long-term natural selection. This allows them to thrive in metal-polluted soils [23]. Due to their ecological and economic value, metallophyte species are considered to be ideal candidates for vegetation restoration and large-scale phytoremediation in metal-contaminated areas [24]. In southwestern China, several pioneer facultative metallophyte plants, such as *Populus yunnanensis* and *Coriaria sinica*, have drawn interest due to their adaptability to environmental stressors, including barren and metal-polluted soils [25,26]. However, the interactions between these pioneer metallophyte species and their root-associated endophytic fungi, including arbuscular mycorrhizal fungi and DSEs, remain poorly understood. We hypothesize that these root-associated endophytes are highly metal-resistant fungi that contribute to their host plants’ adaptability in barren, multi-metal-polluted mine tailing soils. In this study, we assessed the colonization and diversity of AMF and DSEs in the roots of *P. yunnanensis* and *C. sinica* and further investigated the functional roles of DSEs in enhancing Cd tolerance and promoting the growth of host plant under greenhouse conditions.

## 2. Materials and Methods

### 2.1. Study Site and Sample Collection

The study site, Huangmaoshan, is an abandoned tin (Sn), lead (Pb), and zinc (Zn) mine tailing pond located at 103°10′42.2″ E, 23°17′41.7″ N, with an elevation ranging from 1937 to 2460 m above sea level. It is situated in Gejiu City, Yunnan Province, in southwestern China. The region experiences a tropical and subtropical monsoon climate, with a mean annual temperature ranging from 12.5 °C to 14.5 °C and annual precipitation between 200 and 1600 mm. In recent years, *P. yunnanensis*, a species known for its strong adaptation to highly polluted soils in mining areas, was selected for land reclamation. Starting in 2005, *P. yunnanensis* cuttings were planted in the barren slag heaps of the sample areas. Over more than a decade, this species successfully established a dominant community. At the same time, *C. sinica* spontaneously formed a mono-dominant community on slag heaps adjacent to the *P. yunnanensis* plantation. Neither of these two pioneer plant species showed visible symptoms of toxicity or growth defects.

Roots and rhizospheric soils (collected from 10–25 cm depth, totaling more than 500 g) were sampled from 45 *P. yunnanensis* and 35 *C. sinica* plants at the study site. The root samples were divided into two portions: one for evaluating DSE and AMF colonization and assessing the diversity of fungal endophytes using a molecular-based approach, and the other for isolating DSEs. Additionally, biennial branches of *P. yunnanensis* and soil samples were collected for greenhouse plantlet preparation and substrate culture, as described below.

### 2.2. Colonization of DSEs and AMF

For anatomical observation of DSE and AMF colonization, root samples were cleared in 10% (*w*/*v*) KOH at 90 °C for 1.5 h, and then stained with acid fuchsine [27]. The stained roots were sectioned into 2 cm fragments, and at least eight were examined under a compound microscope (OLYMPUS-BX51, Olympus Optical Co., Ltd., Tokyo, Japan) at 400× magnification. Colonization intensity of DSEs (hyphae and intracellular microsclerotia) and AMF (vesicles, arbuscules, running hyphae, and hyphal coils) was assessed by counting over 150 intersections using the magnified intersection method [28].

### 2.3. DSE Isolation and Identification

Fresh root samples of *P. yunnanensis* and *C. sinica* were carefully cleaned to remove any adhering soil particles under running tap water. DSEs were then isolated following the previously described method [29]. Briefly, the root samples were sterilized by immersing them in 75% ethanol for 5 min, followed by 10% NaClO for another 5 min. After sterilization, the roots were rinsed three times in sterile distilled water. The surface-sterile root samples were air-dried under sterile conditions, cut into 3–5 mm pieces, and cultured on malt extract agar (MEA) and potato dextrose agar (PDA) isolation media. For the isolation media, over 80 root pieces from each plant sample were placed on 1% MEA (containing 20 g malt extract, 5 g peptone, 15 g agar, and 1000 mL water, adjusted to a pH of 6.4) and PDA (containing 200 g potato extract, 20 g dextrose, 15 g agar, and 1000 mL water). Both media were supplemented with streptomycin (100 mg L^−1^) and ampicillin (100 mg L^−1^) to prevent bacterial contamination. The cultures were incubated in the dark at 28 °C for 60 days, during which the plates were monitored each day. Newly emerging melanized fungal colonies from the root tissues were transferred to PDA slants for further analysis.

To identify the fungi, the internal transcribed spacer (ITS) rDNA genes (ITS1-5.8S-ITS2) were amplified from all fungal isolates and sequenced. The total genomic DNA of each DSE strain was extracted and purified using the urea extraction method [30]. The ITS1-5.8S-ITS2 regions were amplified using the ITS1-F/ITS4 primer set [31]. Polymerase chain reaction (PCR) was carried out in a total volume of 25 μL, containing 9.5 μL of sterile distilled water, 12.5 μL of TSINGKE Master Mix (TSINGKE, Beijing, China), 1 μL of each 10 μM primer, and 1 μL DNA template. The PCR amplification was performed on a Mastercycler (Eppendorf, Hamburg, Germany) under the following conditions: an initial pre-denaturing at 94 °C for 3 min, followed by 30 cycles of denaturation at 94 °C for 30 s, annealing at 58 °C for 30 s, extension at 72 °C for 100 s, and a final extension at 72 °C for 7 min. The PCR products were electrophoresed on a 1% agarose gel, purified using the Gel Extraction Kit (UNIQ-10, Sangong Biotechnology Co., Ltd., Shanghai, China), and sequenced on an ABI 3730 DNA Analyzer (Applied Biosystems, Inc., Waltham, MA, USA). Endophytic fungal isolates were characterized through phylogenetic analysis of their ITS gene sequences. Operational taxonomic units (OTUs) were clustered using a 99% identity cutoff within the ITS region. Representative sequences from each OTUs were aligned against the UNITE+INSD fungal ITS databases via the BLAST algorithm. Moreover, taxonomic assignments incorporated the digital object identifiers (DOIs) for UNITE fungal species hypotheses set at a 1.5% threshold. For classification at higher taxonomic levels, identity thresholds of 90%, 85%, 80%, and 75% were employed for genus, family, order, and class, respectively [32]. The ITS sequences obtained in this study were deposited in GenBank under accession numbers OP689584–OP689615.

### 2.4. Analysis of Root-Associated Endophytic Fungi by Molecular Approach

Total genomic DNA was extracted from the root samples of *P. yunnanensis* and *C. sinica* (six samples each) using the Magnetic Soil and Stool DNA Kit (Tiangen Biotech Co., Ltd., Beijing, China), following the manufacturer’s protocols. The final DNA concentration and purity were measured using a NanoDrop 2000 UV–vis spectrophotometer (Thermo Scientific, Wilmington, DE, USA), and DNA quality was assessed by 1% agarose gel electrophoresis. Fungal rDNA genes were amplified using the primers ITS1-F (5′-CTTGGTCATTTAGAGGAAGTAA-3′) and ITS2-R (5′-GCTGCGTTCTTCATCGATGC-3′) in a thermocycler PCR system (GeneAmp 9700, Applied Biosystems, Foster City, CA, USA) [33]. The PCR conditions were as follows: initial denaturation at 95 °C for 3 min, followed by 39 cycles of denaturation at 95 °C for 30 s, annealing at 55 °C for 30 s, and elongation at 72 °C for 45 s. A final extension was carried out at 72 °C for 10 min. PCR reactions were performed in triplicate, with each 20 μL reaction mixture containing 2 μL of 10 × FastPfu Buffer, 2 μL of 2.5 mM dNTPs, 0.8 μL of each primer (5 μM), 0.2 μL of TaKaRa rTaq DNA Polymerase, 0.2 μL of BSA, 10 ng of template DNA, and ddH_2_O. The PCR products from each sample replicate were pooled and purified from a 2% agarose gel using the AxyPrep DNA Gel Extraction Kit (Axygen Biosciences, Union City, CA, USA) and quantified using QuantiFluor™-ST (Promega, Madison, WI, USA). Purified PCR products, each containing unique barcodes, were sequenced using the Illumina Miseq platform (Majorbio Bio-Pharm Technology Co., Ltd., Shanghai, China). The raw sequencing data have been deposited in the NCBI Sequence Read Archive (SRA) under accession numbers SAMN31489021–SAMN31489032.

Raw sequence data were pre-treated, including de-multiplexing of barcoded sequences, quality filtering, denoise, chimera checking, and data normalization and referred as clean reads [34,35]; After processing with QIIME (http://qiime.org/, accessed on 16 October 2023) [34], clean sequences with at least 97% similarity were categorized into operational taxonomic units (OTUs) [35]. A Venn diagram showing the distribution of OTUs among different treatments was generated using VennDiagram package of software R (v3.6.1). The community richness index (Chao and ACE estimators) and diversity index (Shannon and Simpson indices) were calculated using QIIME (v 1.9.0) software [34]. The sequenced data were classified into fungal phyla and genera by comparing them with the ITS reference database, Unite [36]. Principal coordinate analysis (PCoA) based on unweighted UniFrac distances was used to describe the differences in fungal community composition in the roots of the two plant species using the R package “PCOA” (v3.6.1) [37]. A heatmap of the Spearman correlation coefficients between environmental factors and fungal aboundance was generated using the pheatmap package in R software (v3.6.1) [38]. All the above data analysis was performed using the free online platform of Majorbio Cloud Platform [39] from Majorbio Bio-pharm Technology Co., Ltd. (Shanghai, China).

### 2.5. Tolerance of Fungal Isolates to Cd Stress

In this study, Cd was chosen as the test heavy metal to evaluate the tolerance of 192 DSE strains by determining their minimal inhibitory concentration (MIC)—the lowest Cd concentration that completely inhibited fungal growth [29,40]. Cd is a non-essential, non-metabolic element with high mobility and a strong tendency to bioaccumulate, making it one of the most toxic heavy metals [20]. Meanwhile, as the primary contaminant in agricultural soils in China, Cd has garnered significant attention [29]. Initially, fungal mycelia were inoculated into a sterile MMN liquid medium, which contained the following components: CaCl_2_ (0.05 g), MgSO_4_ (0.15 g), NaCl (0.025 g), 1% FeCl_3_ (1.2 mL), KH_2_PO_4_ (0.5 g), vitamin B_1_ (100 μg), (NH_4_)_2_HPO_4_ (0.25 g), glucose (15 g), and distilled water to a volume of 1000 mL, with a pH of 5.8. The growth of fungal strains was evaluated in an MMN medium supplemented with increasing concentrations of cadmium (100, 200, 300, 400, and 500 mg L⁻^1^) after a 10-day incubation period at 28 °C. For each Cd concentration, fungal growth was monitored by assessing the emergence of new hyphae from the inoculated fungal discs. If hyphal growth was observed, it indicated that the minimum inhibitory concentration (MIC) for that particular strain was higher than the tested Cd concentration. Conversely, if no hyphal growth occurred, lower concentrations were evaluated to further refine the determination of the MIC. The highest cadmium concentration that inhibited visible growth of the inoculum was recorded as the MIC for each fungal strain. Three replicates were performed for each fungal strain, and a positive control for each fungal isolate was carried out under no cadmium addition. Based on the Cd tolerance and fungal isolation frequencies, six representative DSE strains (*Cladosporium* sp. 4611 and 151 M, *Cyphellophora* sp. 3452, *Leptosphaeria sclerotioides* 6411, *Cadophora luteo-olivacea* 3232, and *Cadophora* sp. 192 M) were selected for further evaluation of their plant growth-promoting traits, including the solubilization of insoluble inorganic/organic phosphorus, production of IAA, and siderophore synthesis.

### 2.6. Determination of Dissolving Phosphorus

The six representative DSE strains were selected to assess their abilities to dissolve organic phosphorus (phytin) and inorganic phosphorus (tricalcium phosphate, TCP), both of which are typically unavailable to plants. Fungal isolates stored at −86 °C were inoculated on a PDA medium and cultured at 28 °C for two weeks. Then, mycelial inoculum discs were aseptically cut out from the margins of actively pre-grown cultures, and two discs were inoculated into a 250 mL conical flask, containing 100 mL of PVK liquid medium. The medium composition was as follows: glucose (10 g), (NH_4_)_2_SO_4_ (0.5 g), MgSO_4_ (0.3 g), KCl (0.3 g), FeSO_4_ (0.03 g), NaCl (0.3 g), MnSO_4_ (0.03 g), insoluble phosphorus source (5 g), distilled water to make up to 1000 mL, and adjusted to a pH of 6.5. Three replicates were conducted for each fungal strain. The inoculated flasks were then incubated at 28 °C with shaking at 180 rpm for 10 days. After incubation, the fungal biomass was filtered and collected, rinsed three times with sterile distilled water, and weighted. Dry weight was determined by drying the fungal biomass in an oven at 60 °C until a constant final weight was achieved (48 h). The filtered culture medium was used to measure the pH value using a pH meter (FE20 Plus, METTLER TOLEDO, Zurich, Switzerland). The final concentration of soluble phosphorus in the medium was determined using the molybdenum antimony anti-colorimetry method [41,42].

### 2.7. Determination of Siderophore Production

The siderophore production of the six DSE strains was evaluated using the chrome azurol S (CAS) agar plate assay, following the method described by Milagres et al. [43]. A CAS-blue solution was prepared by combining the first solution (solution A), which consisted of 60.5 mg CAS dissolved in 50 mL deionized water and 10 mL of Fe^3+^ solution (1 mM FeCl_3_·6H_2_O in 10 mM HCl), with solution B, containing 72.9 mg HDTMA (Hexa-decyl Trimethyl Ammonium bromide) dissolved in 40 mL deionized water. This mixture was filter-sterilized using 0.22-μm filters. Next, 10 mL of the CAS-blue solution was mixed with 100 mL of 1% agar, which had been pre-autoclaved at 121 °C for 15 min and cooled to approximately 60 °C. 15 mL of the CAS medium was poured into sterile 9 cm Petri plates. Once the medium solidified, approximately 15 mL of an iron-free MMN medium (containing 0.05 g CaCl_2_, 0.15 g MgSO_4_, 0.025 g NaCl, 0.5 g KH_2_PO_4_, 100 μg Vitamin B_1_, 0.25 g (NH_4_)_2_HPO_4_, 15 g glucose, 10 g agar, 1000 mL deionized water, autoclaved at 121 °C for 15 min) was also added to the above prepared petri dish, creating CAS-MMN double-layer plates. Then, each fungal strain was inoculated onto the double-layered plates and incubated in darkness at 28 °C for 8 days. Negative controls without fungal inoculation were also included. After the incubation period, color changes around the fungal colonies were observed and recorded.

Each fungal strain was inoculated into a 250 mL conical flask containing 100 mL of the iron-free MMN liquid medium and incubated at 28 °C with shaking at 180 rpm. Simultaneously, negative controls without fungal inoculation were prepared. Each treatment was conducted in triplicate. After 5 days of incubation, 5 mL of the cultured media was filtered through a 0.22 μm filter membrane and then mixed with 5 mL of CAS dye solution. This mixture was incubated statically at room temperature for 1 h. The optical density (OD) of the samples was then measured at a wavelength of 680 nm using a UV–vis spectrophotometer (Model 752N, INESA Scientific Instrument Co., Ltd., Shanghai, China). The siderophore production was quantified as siderophore units (SUs) using the following formula:

$$\%Siderophore\;units\;(SUs)=\frac{Ar-As}{Ar}\times 100$$

where Ar is the absorbance of the negative control, and As is the absorbance of the sample, both measured at 680 nm.

### 2.8. Determination of Indole-3-Acetic Acid (IAA)

Indole-3-acetic (IAA) production by the six DSE strains was evaluated using LC-MS methods. The fungal isolates were first incubated in 200 mL of Potato Dextrose Broth (PDB) supplemented with 1 mg mL^−1^ tryptophan for 10 days at 28 °C in darkness, with shaking at 200 rpm. Each fungal isolate was tested in triplicate. Following incubation, the culture broth was extracted twice with ethyl acetate at a 1:1 (*v*/*v*) ratio. The extraction process involved 20 min of ultrasonic vibration treatment, followed by stationary incubation overnight at room temperature. The ethyl acetate-extracted fraction was then collected and dried using a rotary vacuum evaporator (Eyela OSB-2100, Tokyo Rikakikai Co., Ltd., Tokyo, Japan). The dried extract was dissolved in acetone and re-evaporated three times to remove the residual solvent. The concentrated extract was then dissolved in 300 mL of methanol and stored at −20 °C.

High-performance liquid chromatography–mass spectrometry (LC-MS) was used to evaluate the concentration of IAA produced by fungal isolates using a Q Exactive^TM^ focus system (Thermo Fisher Scientific, Bremen, Germany). A 10 μL aliquot of each sample was injected into the analytical column and maintained at 30 °C. The elution was carried out using an acetonitrile–water system at a flow rate of 1 mL min^−1^. IAA quantification was performed by comparing the peak areas of the samples to those of authentic IAA standards (Yuanye Bio-Technology Co., Ltd., Shanghai, China) with known concentrations.

### 2.9. Alleviation of Metal Stress by DSEs in P. yunnanensis

Biennial branches of *P. yunnanensis* were collected from the sample site, cleaned under running tap water, and cut into 15 cm segments. The segments were surfaced-sterilized by soaking in 10% sodium hypochlorite for 5 min and rinsed three times with sterile water. The sterilized cuttings were then soaked in a 1 mg/L NAA solution for 24 h as a pre-treatment. Following pre-treatment, the cuttings were transferred to sterile water and cultured in a greenhouse to promote rooting and sprouting under natural lighting at a day/night temperature of 25 ± 3 °C/22 ± 3 °C for 60 days. The water was renewed every two days. After 60 days, plantlets of similar size were selected for inoculation with *Cyphellophora* sp. 3452 (isolated from *P. yunnanensis*) and *Cadophora* sp. 192M (isolated from *C. sinica*), respectively, following the method described by Wang et al. [17]. Negative controls were inoculated with the same quantity of sterile fungal inoculants that had been autoclaved at 121 °C for 15 min. All plantlets, including controls, were cultured in plastic pots (120 mm top diameter, 80 mm base diameter, 153 mm height) containing 1.5 kg of tailing soils collected from the sampling sites. The soils were autoclaved at 121 °C for 120 min for three cycles with two-day intervals. The plantlets were watered three times a week with deionized water and once a week with 30 mL of 1/2 Hoagland’s solution. After 60 days of greenhouse culture, the net height and fresh weight of each plantlet were measured by subtracting the initial biomass at the time of planting from the fresh weight of the harvested samples. Additionally, the presence of the two DSE inoculants within the poplar roots, including melanized septate hyphae and microsclerotia, was examined and measured in poplar roots using microscopy, following the methods described above. The roots, stems, and leaves were separately collected and dried to a constant weight at 60 °C. Each dry sample was ground, and 0.5 g of the ground sample was wet-digested using a nitric acid–perchloric acid mixture. The concentrations of Pb, Zn, and Cd in the samples were determined using a flame atomic absorption spectrometer (ZA3000, HITACHI, Tokyo, Japan) as previously described [17]. Translocation factors (TF) were calculated as the ratio of the concentrations of heavy metals in the stems and leaves to the actual measured root metal concentrations. Additionally, rhizosphere soil was collected and air-dried. A 5.0 g sample of soil was mixed with 50 mL of 0.1 M HCl solution and incubated at 200 rpm at 20 °C for 2 h. After filtration, the concentrations of 0.1 M HCl-extractable Pb, Zn, and Cd, which may reflect bioavailable fractions of metals [44], were determined. The bioconcentration factors (BCF) of heavy metals in *P. yunnanensis* were calculated as the ratio of the concentrations of heavy metals in the roots to the 0.1 M HCl-extractable metal concentrations in the soil. The effect of fungal inoculation on poplar growth and metal accumulations was assessed using the fungal inoculation effect (FE) formula:

$$FE=\frac{T^{+}-T^{-}}{T^{-}}$$

where T^+^ and T^−^ represent the DSE-inoculated treatments and uninoculated controls, respectively, as described by Yin et al. [45].

### 2.10. Statistical Analysis

Statistical analyses were performed using IBM SPSS Statistics 25 software (SPSS Inc., Chicago, IL, USA), and graphical presentations were created using Origin 2018 software (OriginLab, Los Angeles, CA, USA). A Student’s t-test was employed to reveal significant differences in the colonization rate, richness indices, and diversity indices of fungal communities between the roots of the two plant species, as well as in the biomass of the experimental and control groups in the greenhouse experiment. To evaluate mycelial functions, including phosphorus-dissolving capacity and IAA concentrations, a one-way ANOVA followed by Duncan’s multiple-range test was conducted. Statistical significance was set at *p* < 0.05.

## 3. Results

### 3.1. Colonization of the Two Plant Roots by AMF and DSEs

Under compound microscopy (BX51, Olympus Optical Co., Ltd., Tokyo, Japan), diverse morphological structures of DSEs and AMF were observed in the root samples of *P. yunnanensis* and *C. sinica* (Figure S1). The total colonization rate of both DSE and AMF in the roots of *P. yunnanensis* was significantly higher than in *C. sinica* (*p* < 0.05). In the 10 samples investigated, DSE and AMF colonization rates in *P. yunnanensis* were 73.49 ± 2.96% and 66.72 ± 1.57%, respectively, compared to 37.44 ± 0.76% and 54.66 ± 0.81% in *C. sinica*. Notably, *C. sinica* roots exhibited significantly higher AMF colonization than DSE colonization (*p* < 0.001), while *P. yunnanensis* roots showed the opposite trend (Table 1).

### 3.2. Diversity of Culturable DSEs in the Roots of Two Pioneer Plant Species

A total of 192 fungal isolates were obtained, including 104 strains from *P. yunnanensis* and 88 strains from *C. sinica*. The phylogeny of internal transcribed spacer (ITS) rDNA sequences (ITS1-5.8S-ITS2) showed that these isolates belonged to the fungal members of 20 genera and Herpotrichiellaceae (Table 2). Among these culturable root-associated endophytic fungi, five were dominant, each comprising more than 5% of the isolates. The largest *Cadophora,* consisted of 68 fungal isolates (35.42%), followed by *Cladosporium* (44 strains, 22.92%), *Cyphellophora* (15 strains, 7.81%), members belonging to Herpotrichiellaceae (11 strains, 5.73%), and *Paraphoma* (10 strains, 5.21%). Other clades, including *Pyrenochaeta* (5 strains, 4.69%), had a relative abundance of less than 5% (Table 2).

### 3.3. Diversity of Root-Associated Endophytic Fungi in the Roots of the Two Plant Species

A total of 733,197 raw sequences were obtained from the 12 samples, with an average valid sequence length of 265 bp. The rarefaction curves gradually saturated with increasing Illumina sequences, indicating that our sequencing data sufficiently covered the dominant fungal species colonizing the roots of both plant species (Figure S2). The richness indices (ACE and Chao) of fungal communities colonizing *P. yunnanensis* roots were significantly higher than those for *C. sinica* (*p* < 0.05), although no significant difference was observed in the fungal diversity indices (Shannon and Simpson) between the two plant species (Table 3). Overall, 291 fungal operational taxonomic units (OTUs) were detected in the roots of *P. yunnanensis* and 179 OTUs in the roots of *C. sinica* (Figure S3). Only 73 OTUs were shared between the two species, representing 25.09% of the total OTUs in *P. yunnanensis* and 40.78% in *C. sinica*. In contrast, *P. yunnanensis* had 218 specific OTUs (74.91%), while *C. sinica* had 106 specific OTUs (59.22%). Both values were significantly higher than the number of shared OTUs (Figure S3).

**Figure 1:**
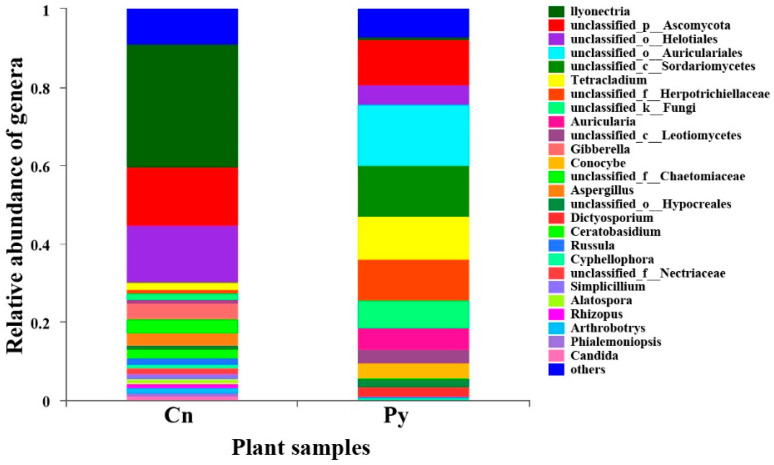
Relative abundance of fungal genera colonizing the roots of *P. yunnanensis* (Py) and *C. sinica* (Cn) in the abandoned tailing area of Huangmaoshan, Yunnan Province, southwest China.

**Figure 2:**
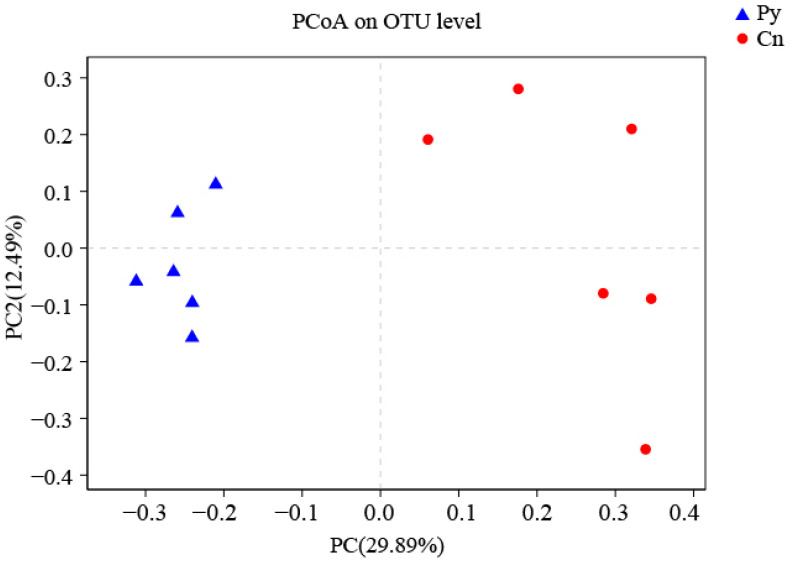
PCoA (unweighted UniFrac) analysis of fungi colonizing the roots of *P. yunnanensis* and *C. sinica* in the abandoned tailing area of Huangmaoshan, Yunnan Province, southwest China.

The compositions of genera in the fungal communities colonizing the two host species were different (Figure 1). Although *C. sinica* hosted a higher number of genera with over 1% abundance (18 genera) compared to *P. yunnanensis* (12 genera), only three dominant genera in *C. sinica*—*Ilyonectria*, unclassified_p_Ascomycota, and unclassified_o_Helotiales—belonged to the Ascomycetes and had proportions exceeding 5%. In contrast, *P. yunnanensis* had eight dominant fungal genera, including five Ascomycetes genera: unclassified_c_Sordariomycetes, *Tetracladium*, uniclassified_f_Herpotrichiellaceae, and two genera that overlapped with *C. sinica* (unclassified_p_Ascomycota and uniclassified_o_Helotiales). Additionally, *P. yunnanensis* hosted two basidiomycete genera (uniclassified_o_Auriculariales, and *Auricularia*) and one unclassified fungal genus (unclassified_k_Fungi). The genus *Ilyonectria* was the most abundant in *C. sinica*, making up 31.57% of the total fungal community. In contrast, its presence in *P. yunnanensis* was drastically lower, representing only 0.77% of the total fungal abundance. This distinct difference in fungal community composition between the two plant species was further supported by PCoA analysis using unweighted UniFrac distances (Figure 2).

**Figure 3:**
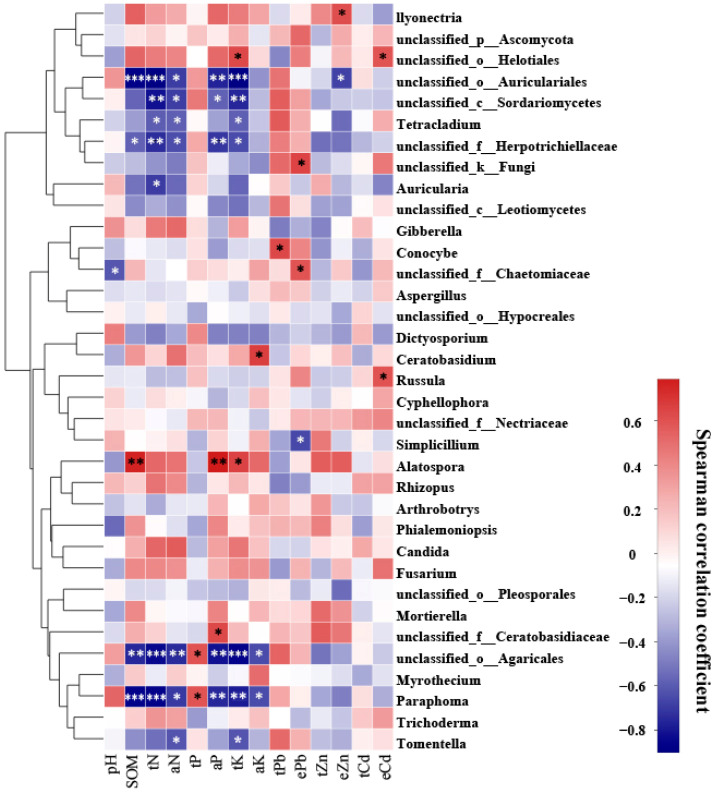
Heatmap showing the correlation between 14 environmental factors and the top 35 most abundant fungal genera colonizing the roots of *P. yunnanensis* and *C. sinica* in the abandoned tailing area of Huangmaoshan, Yunnan Province, Southwest China. Environmental factors include pH, soil organic matter (SOM), total nitrogen (tN), total phosphorus (tP), total potassium (tK), available N (aN), available phosphorus (aP), available potassium (aK), total lead (tPb), total zinc (tZn), total cadmium (tCd), and extractable metals (ePb, eZn, eCd). Spearman’s correlation coefficient was deemed statistically significant at the levels of 0.05 (*), 0.01 (**), and 0.001 (***), respectively.

A Spearman correlation analysis was performed to examine the relationship between 14 environmental factors and the most abundant 35 fungal taxa colonizing the roots of *P. yunnanensis* and *C. sinica* (Figure 3). The results indicated that soil chemical properties influenced the fungal community structure. In particular, total potassium (K) was identified as a significant environmental factor influencing the relative abundance of the nine fungal genera in the plant roots, followed by total nitrogen, available nitrogen, and available phosphorus. We also observed that only a few fungal taxa were closely associated with soil total lead (Pb) concentrations (1 taxon), HCl-extractable Pb (3 taxa), Zinc (Zn) (2 taxa), and cadmium (Cd) (2 taxa).

### 3.4. Cadmium Tolerance of Culturable Root-Associated Endophytic Fungi

**Figure 4:**
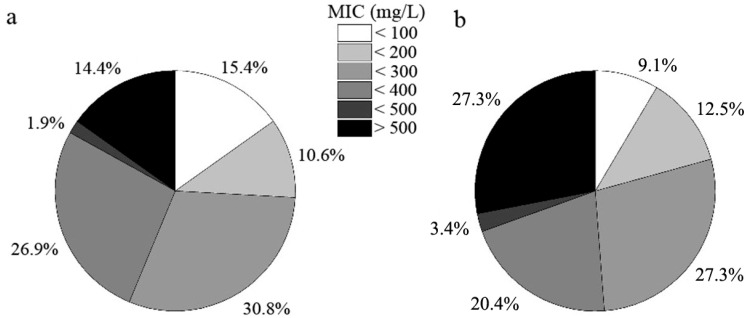
Tolerance of DSE strains colonizing the roots of *P. yunnanensis* (**a**) and *C. sinica* (**b**) to Cd^2+^ as determined by the minimum inhibitory concentration (MIC) range in the abandoned tailing area of Huangmaoshan, Yunnan Province, southwest China.

Root-associated DSE strains exhibited distinct tolerance to varying levels of Cd stress (Figure 4). Approximately 9.1% of the fungal strains (eight strains) isolated from *C. sinica* could not survive under 100 mg L^−1^ Cd, while this number was higher in *P. yunnanensis* (15.4%, 16 strains). The proportion of highly tolerant DSE strains, defined as those with a minimum inhibitory concentration (MIC) above 500 mg L^−1^ Cd, was significantly greater in *C. sinica* (27.3%, 24 strains) compared to *P. yunnanensis* (14.4%, 15 strains).

### 3.5. Phosphorus Solubilizin Ability of the Six Representative DSE Strains

**Figure 5:**
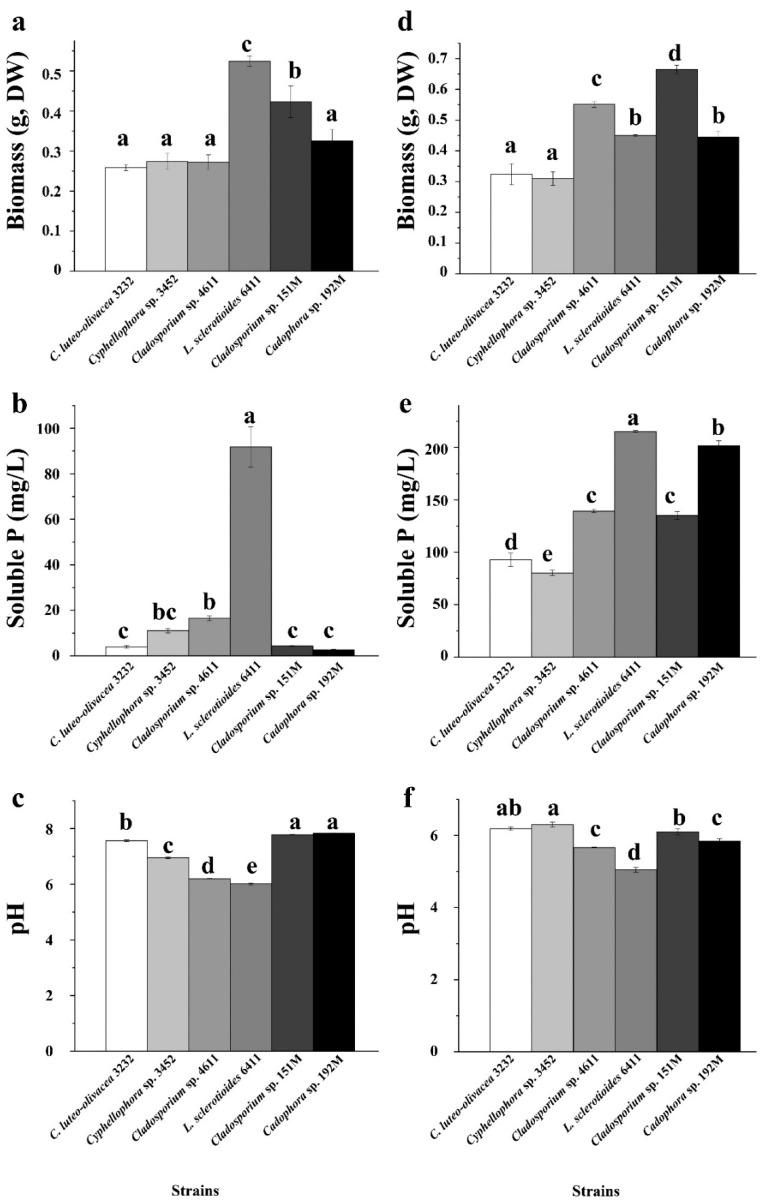
Phosphorus solubilizing capacity of the six representative DSE strains isolated from the roots of *P. yunnanensis* and *C. sinica* in the abandoned tailing area of Huangmaoshan, Yunnan Province, Southwest China. The strains were cultured in a PVK liquid medium supplemented with tricalcium phosphate (**a**–**c**) and Phytin (TCP) (**d**–**f**) as the sole phosphorus source for 10 days. (**a**,**d**) Dry weight of DSE strains; (**b**,**e**) concentration of soluble P in PVK liquid medium; (**c**,**f**) pH value in PVK liquid medium. Different lowercase letters indicate significant differences among different DSE strains (one-way ANOVA, Duncan’s multiple range test, *p* < 0.05).

In our experiment assessing phosphorus-dissolving capacity, we found that the biomass of the six DSE strains was generally higher when the culture medium was supplemented with insoluble organic phytin compared to inorganic tricalcium phosphate (TCP) (*p* < 0.05), with the exception for the strains *C. luteo-olivacea* 3232 and *Cyphellophora* sp. 3452 (Figure 5). This pattern aligned with the concentrations of available phosphorus in the culture broth. The six DSE strains exhibited varying abilities to dissolve different types of insoluble phosphorus. Strains *L. sclerotioides* 6411 and *Cladosporium* sp. 151M showed a relatively high utilization of insoluble inorganic phosphorus, achieving significantly greater biomass than the other four strains. However, when supplemented with insoluble organic phytin, strain *L. sclerotioides* 6411 produced only moderate biomass among the six, while *Cladosporium* sp. 151M maintained the highest biomass, followed by *Cladosporium* sp. 4611 and *Cadophora* sp. 192M. Strains *C. luteo-olivacea* 3232 and *Cyphellophora* sp. 3452 exhibited the least growth. Additionally, we observed a similar trend in the pH of the culture broth, with a generally lower pH in the medium supplemented with insoluble phytin compared to TCP (Figure 5). This decrease in pH may contribute to the strains’ phosphorus-dissolving abilities.

### 3.6. Siderophore Production of the Six Representative DSE Strains

The CAS plate experiments revealed that the six DSE strains exhibited varying abilities to produce and excrete siderophores, as indicated by the color change of the CAS dye from blue to purple or purple–red (Table 4, Figure S4). DSE strains *L. sclerotioides* 6411, *Cladosporium* sp. 4611, and *Cladosporium* sp. 151M were identified as high siderophore producers, with SU values exceeding 0.5 and As/Ar values below 0.5. Among these, strain *L. sclerotioides* 6411 demonstrated the strongest siderophore production, as confirmed by both the CAS dye results and siderophore activity assays (Table 4, Figure S4).

### 3.7. Indole-3-Acetic Acid (IAA) Production of the Six Representative DSE Strains

**Figure 6:**
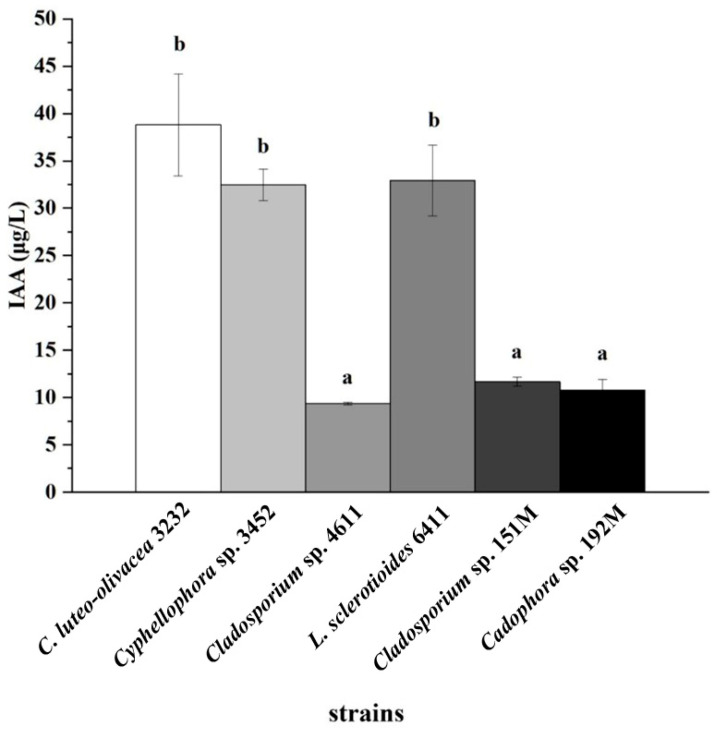
Indole-3-acetic acid (IAA) concentrations in the culture filtrates of six representative DSE strains after 10 days of incubation with L-tryptophan. Different lowercase letters indicate significant differences among the different DSE strains (one-way ANOVA, Duncan’s multiple range test, *p* < 0.05).

The experiment on IAA production showed that all six DSE strains were capable of producing IAA (Figure S5). Strain *C. luteo-olivacea* 3232 produced the highest concentration of IAA, followed by strains *Cyphellophora* sp. 3452 and *L. sclerotioides* 6411, which had a concentration of 38.8 ± 5.39 μg L^−1^. This concentration significantly surpassed that of the other three DSE strains (Figure 6).

### 3.8. The Effect of DSE Inoculation on the Growth of P. yunnanensis

**Figure 7:**
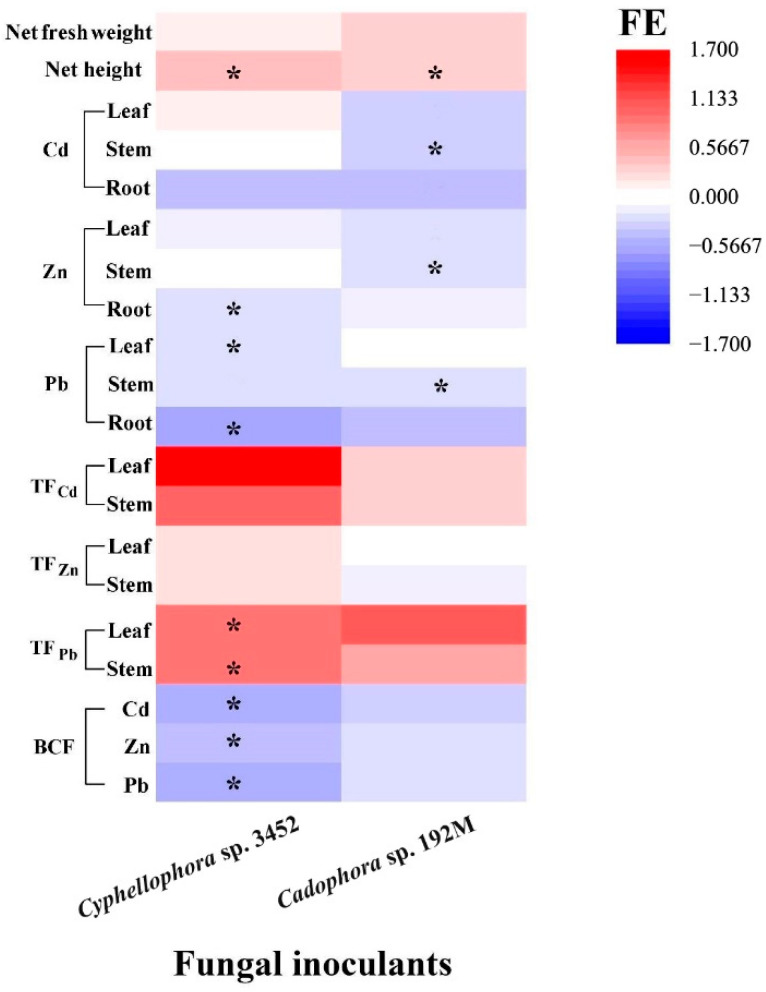
Effects of inoculation by the two fungal strains (FE) on the growth and heavy metal concentrations (Cd, Zn, and Pb) accumulated in the roots, stems, and leaves, as well as on the translocation factors (TF) and bioconcentration factors (BCF) of *P. yunnanensis* inoculated with different DSE strains, compared to non-inoculated controls after 60 days of cultivation. Different lowercase letters indicate significant differences among the different treatments (one-way ANOVA, Duncan’s multiple range test, *p* < 0.05). Asterisks indicate significant differences between the DSE inoculation treatments and their respective uninoculated controls (* *p* < 0.05, *t*-test).

In the greenhouse, we assessed the inoculation effects of one of the two most frequently isolated dominant DSE strains on the growth and metal accumulation in *P. yunnanensis* over a period of 60 days. It was found that two strains (*Cyphellophora* sp. 3452 and *Cadophora* sp. 192M) developed typical colonization structures, including microsclerotia and septate hyphae, in the roots of *P. yunnanensis* (Figure 7 and Figure S6), and the other four strains failed to form these structures. The colonization rates in the roots were 41.8 ± 3.60% for stain *Cyphellophora* sp. 3452 and 49.2 ± 2.17% for strain *Cadophora* sp. 192M. Colonization by these two DSE strains alleviated the toxicity of excessive soil metals and promoted the growth of the host plant (Figure S6). *P. yunnanensis* inoculated with DSEs showed a significant increase in height compared to the uninoculated controls (*p* < 0.05), and while total biomass (fresh weight) also increased, this difference was not statistically significant (*p* > 0.05). Our results further indicated that DSE inoculation influences the uptake of metal ions, altering the distribution of metals in different organs (stems and leaves) (Figure 7). Compared to the uninoculated controls, DSE strain *Cadophora* sp. 192M significantly reduced the accumulation of Pb, Zn, and Cd in the stems of *P. yunnanensis*. Similarly, inoculating *Cyphellophora* sp. 3452 markedly inhibited Zn and Pb uptake in the roots and Pb accumulation in the poplar leaves. Consequently, the bioconcentration factor for these metals decreased in DSE strain *Cyphellophora* sp. 3452-inoculated plants. DSE inoculation altered metal distribution within the plant. DSE strain *Cyphellophora* sp. 3452 led to a significant increase in Pb translocation factors in leaves and stems compared to the uninoculated controls. In contrast, DSE strain *Cadophora* sp. 192M resulted in only a slight, non-significant increase in metal translocation factors in leaves and stems (*p* > 0.05) (Figure 7).

**Figure 8:**
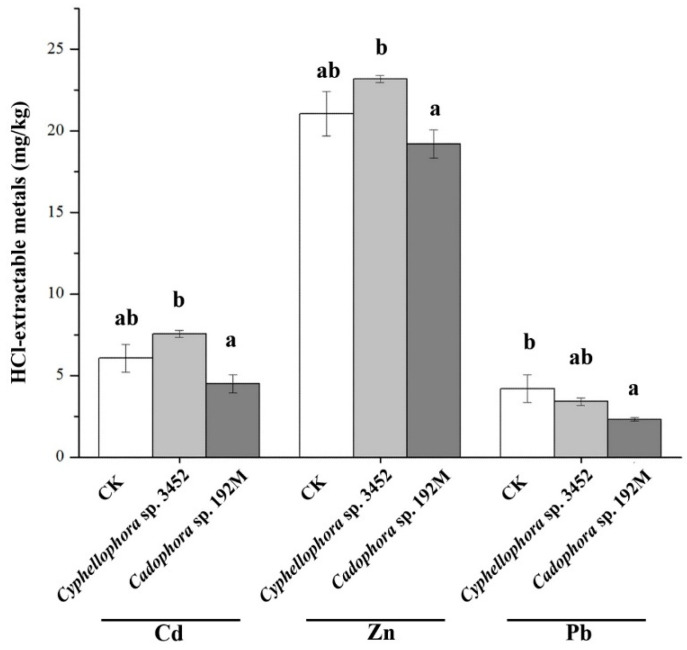
Concentrations of HCl-extractable metals in the rhizosphere soil after 60 days of cultivation of *P. yunnanensis.* Different letters within the same heavy metal group indicate significant differences between the treatment groups (one-way ANOVA, Duncan’s multiple range test, *p* < 0.05).

Furthermore, plants inoculated with the two DSE strains (*Cyphellophora* sp. 3452 and *Cadophora* sp. 192M) have different effects on the bioavailability of metals in soil, varying for both the fungal inoculants and the type of metal (Figure 8). We found that the plant inoculated with DSE decreased the metal availability in soil, particularly with lower concentrations of HCl-extractable Pb in the rhizosphere compared to the uninoculated control (*p* < 0.05). In contrast, inoculation with strain *Cyphellophora* sp. 3452 resulted in a slight increase in the availability of Cd and Zn in the soil, although these changes were not statistically significant (*p* > 0.05), (Figure 8).

## 4. Discussion

As dominant root colonizers, both arbuscular mycorrhizal fungi (AMF) and dark septate endophytes (DSEs) are widely found in the roots of various plant species globally, including those in metal-polluted soils like mine tailings [18,46]. In this study, we observed that these two types of root fungal colonizers abundantly colonized the roots of two pioneer tree species. Notably, both fungal groups showed a preference for colonizing poplar roots, with significantly higher colonization intensity compared to *C. sinica*. This finding is consistent with previous reports on host preference for root fungal colonizers [11,47]. For example, Yin et al. [45] found that different maize cultivars with distinct genetic traits exhibited varying levels of mycorrhizal compatibility with *Funneliformis mosseae.* Additionally, Loo et al. [48] discovered that axial differentiation of root segments within the same plant root can influence spatial colonization by microbiota along the root axis. This preferential colonization is shaped by a complex interplay of factors, including host plant identity, environmental conditions, and microbial type [1,48]. For example, Jones and French [49] found that AMF colonization rates were lowest under high-nutrient conditions but increased when nitrogen or phosphorus was limited, whereas DSE colonization was higher in nutrient-rich soils. Furthermore, the varying dependence of different plant species on root-associated fungi under heavy metal stress may also contribute to distinct colonization patterns [50,51]. Given the known roles of root-associated fungi, it is reasonable to conclude that the regulation of fungal colonization and community composition by plants results from the adaptive interaction between plants and endophytic fungi that have co-evolved with their environment [18,52].

Our research revealed that the roots of the two pioneer plants in abandoned heavy metal tailing soil harbored a rich and diverse community of endophytic fungi. In heavily contaminated mine soils, severe metal pollution and intense mining and smelting activities have caused significant structural degradation of the tailing soil and reduced biological activity. These factors present key barriers to the natural succession of vegetation in such environments [53,54]. However, pioneer plants colonizing these mine tailings can adapt to these stressful conditions by developing tolerant functional traits and recruiting specific soil microbial communities, as well as mycobiome, which act as both followers and facilitators in restoration ecology [55,56]. Our study also found significant differences in the endophytic fungal communities between the roots of the two pioneer plants. This finding is consistent with Gagnon et al. [57], who observed that the boreal species used for reclamation, such as Speckled alder (*Alnus incana* ssp. *rugosa*), paper birch (*Betula papyrifera*), and spruce (*Picea* sp.), are key drivers of microbial population composition in their bulk soil, rhizosphere, and root endosphere in the northwestern region of Québec (Canada), known for its gold deposits. Under both biotic and abiotic stress conditions, plants can modify their exudation patterns to selectively recruit a beneficial ‘stress-tolerant microbiome’, enabling them to better cope with harsh environmental conditions [58,59]. Additionally, we isolated a diverse and abundant resource of endophytic fungi from the roots of both pioneer plants in the mining areas, with significantly more strains isolated from *P. yunnanensis* compared to *C. sinica* (104 strains vs. 88 strains). This result is consistent with the higher fungal richness indices (ACE and Chao) observed in *P. yunnanensis* compared to *C. sinica*. Numerous studies have also reported that poplar species harbor a rich diversity of fungal symbionts in their roots, including dual mycorrhizal associations with ectomycorrhizal (ECM) and arbuscular mycorrhizal (AM) fungi, as well as non-mycorrhizal fungal endophytes such as DSEs and other fungal endophytes [40,60]. Additionally, we also noticed that *P. yunnanensis* hosted two basidiomycete genera (uniclassified Auriculariales, and *Auricularia*). In contrast, there have been fewer reports on root-associated endophytic fungi in *C. sinica* [61], with more focus on nitrogen-fixing endophytes such as *Frankia* [62]. Both species are pioneer plants capable of thriving in heavy metal-contaminated tailing soils without exhibiting symptoms of heavy metal toxicity [63,64]. We identified *Ilyonectria* as the dominant genus within the root endophytic fungal community of *C. sinica*, comprising 35.57% of the total community. In contrast, *Ilyonectria* represented only 0.77% of the endophytic fungal community in the roots of *P. yunnanensis*. These findings suggest that different plant species recruit distinct root endophytic fungal communities to better adapt to environmental stresses. It is hypothesized that these spontaneously reclaimed root-associated endophytic fungi may play a crucial role in enhancing the adaptability of these hosts to tailing-stressed soils through natural succession.

Our research demonstrates that all six representative strains of DSE fungi possess plant growth-promoting capabilities, although the extent of these effects varies among the strains. This finding is similar to those of Soto et al. [65], who observed that DSE strains colonizing the roots of native Ericaceae plants in the volcanic deposits of the Andes Mountains, southern Chile, exhibited different plant growth-promotion traits and high in vitro tolerance to abiotic stress, such as aluminum and water stress. Our study revealed that all six DSE strains were capable of solubilizing both insoluble inorganic phosphate (TCP) and organic phosphate (calcium phytate) in vitro, consistent with previous research. For example, *Phialophora fortinii* has been shown to degrade various carbon and phosphorus compounds, including cellulose, starch, lipids, casamino acids, gelatin, urea, and pectin [66]. Similarly, Spagnoletti et al. [67] discovered that nine DSE fungi, including *Ophiosphaerella herpotricha* and *Drechslera* sp., can solubilize insoluble inorganic phosphates such as calcium, aluminum, and iron phosphates. We also found that DSE strains exhibited a higher solubilization capacity for organic phosphate (calcium phytate) than for inorganic tricalcium phosphate. This finding supports the notion that DSE fungi are more commonly found in soils with high organic matter content [68,69] and are more efficient at mineralizing organic phosphorus [70,71].

Importantly, once DSE fungi colonize plant roots, they can establish a symbiotic relationship with the host plant, similar to that formed by mycorrhizae [3]. DSEs secrete a variety of enzymes that mineralize insoluble phosphorus in the soil, converting it into soluble forms and thereby enhancing the interaction between plants and soil [70,71]. In this study, all six representative DSE strains exhibited a strong capacity to solubilize organic phosphorus, with *L. sclerotioides* 6411 showing a relatively higher ability to dissolve inorganic tricalcium phosphate. These findings suggest that phosphorus solubilization is a key factor by which root endophytic fungi enhance host plant tolerance to environmental stress and promote plant growth. The colonization of roots by these fungi significantly increases the activities of enzymes such as acid phosphatase, alkaline phosphatase, and urease, thereby improving phosphorus availability and the phosphorus pool in the rhizosphere. As a result, this enhances phosphorus nutrition and promotes plant growth in host plants such as blueberries [71,72]. By converting insoluble phosphorus into soluble forms, DSEs facilitate the absorption and utilization of phosphorus by the host plant, ultimately increasing its biomass [14,65]. Additionally, the phosphorus-solubilizing capabilities of these naturally reclaimed and successional root-associated endophytic fungi also contribute to soil remediation [73]. The solubilized phosphorus can also act as a soil amendment, chelating heavy metal ions in the soil, reducing the bioavailability of toxic metals, and enhancing the phytostabilization function of the rhizosphere, thus strengthening the host plant’s ability to remediate contaminated soils [74].

Furthermore, all six DSE strains in our study demonstrated the ability to produce indole-3-acetic acid (IAA), with strains *Cladosporium* sp. 4611, *Cladosporium* sp. 151M, and *L. sclerotioides* 6411 showing high capacities for siderophore production. These traits contribute to their plant growth-promoting properties [40]. The auxins and other plant hormones secreted by DSE fungi can modify the root architecture of the host plant, increasing the root surface area and enhancing water and nutrient uptake, which promotes plant growth and can even facilitate the colonization of the roots by other fungi [75]. Additionally, siderophores, acting as metal chelators, not only supply iron to plants but also aid in the absorption and transport of heavy metals, improving resistance to heavy metal stress for both the fungi and the host plants [76].

Greenhouse inoculation experiments with two representative DSE strains showed that DSE inoculation alleviated the toxic effects of excessive heavy metals on the host plants. This was achieved by reducing metal uptake by the roots and limiting its translocation to aboveground parts, thereby promoting plant growth. These results align with previous studies, which suggest that DSE colonization in tailing soils plays a crucial role in the natural revegetation process for pioneer plants [7]. DSEs may be essential to the functioning of metal-contaminated ecosystems, improving the ability of host plants to withstand stress in tailing soils [7]. Research has shown that, like AMF, DSEs are dominant fungal colonizers in the roots of many plants in tailing reclamation, with strong tolerance to heavy metals [10]. Unlike AMF, which often decrease in root colonization or disappear as pollution intensifies, DSEs maintain their dominance in the roots of pioneer plants under severe stress in tailing soils [21].

Our previous research has demonstrated that DSEs can enhance host plant resistance to heavy metals by increasing their ability to chelate toxic metal ions, promoting their transformation of these ions into less toxic forms, and activating the host plant’s antioxidant defenses [17]. Additionally, DSEs increase the greater abundance of functional groups in pectin and hemicellulose 1, such as carboxyl groups, in response to Cd stress. These functional groups provide more binding sites for Cd, strengthening the fixation and compartmentalization of metals within the remodeled cell wall matrix. This reduces metal accumulation in critical organelles, thereby mitigating toxicity [77]. Given their plant growth-promoting properties, versatility, and ease of cultivation, DSEs are promising candidates for enhancing phytoremediation and revegetation efforts in heavy metal-contaminated, saline, and decertified lands [7].

## 5. Conclusions

In conclusion, a variety of endophytic fungi, including common root symbionts like AMF and DSE fungi, naturally colonized the roots of two pioneer tree species during the revegetation of heavily metal-polluted slag heaps. However, the two plant species exhibited distinct preferences for recruiting different root-associated endophytic fungi, leading to the formation of compositionally distinct fungal communities. Our findings suggest that these root-associated endophytic fungi possess diverse plant growth-promoting traits in vitro. When inoculated into the roots of *P. yunnanensis*, they have the potential to reduce the phytotoxic effects of excessive metals in the soil, playing a crucial role in maintaining plant root health. Our study emphasizes the diverse functional properties of different DSE strains, highlighting the need for further research on how host plants adapt through the beneficial interactions with these strains. Our findings suggest that utilizing root-associated fungi can serve as an alternative or complement to traditional revegetation and reclamation methods—such as topsoil application, fertilizers, or hydraulic seeding [78]—in restoring severely metal-contaminated slag heaps. This approach provides an economically feasible and ecologically sustainable solution for restoring large-scale, highly polluted mining sites, including those with *P. yunnanensis*. Additionally, by engineering or manipulating microbiome–plant systems, we can enhance soil restoration efforts, maximizing both economic and ecological benefits while minimizing risks. This strategy underscores the importance of plant–microbe synergies in remediating metal-contaminated environments.

## Figures and Tables

**Table 1:** The colonization rates of dark septate endophytes (DSEs) and arbuscular mycorrhizal fungi (AMF) in the roots of *P. yunnanensis* and *C. sinica* in the abandoned tailing area of Huangmaoshan, Yunnan Province, southwestern China. The rates are presented as means ± SE (*n* = 10). Different lowercase letters within each row indicate significant differences in fungal colonization rates between *P. yunnanensis* and *C. sinica* (*t*-test, *p* < 0.05).

Fungal Structures	*P. yunnanensis*	*C. sinica*
DSEs		
Hyphae	72.2 ± 3.0% a	36.6 ± 0.8% b
Microsclerotia	1.3 ± 0.2% a	1.5 ± 0.5% a
Total	73.5 ± 3.0% a	37.4 ± 0.8% b
AMF		
Hyphae	55.8 ± 1.2% a	49.5 ± 0.7% b
Arbuscules	5.4 ± 0.6% a	3.0 ± 0.5% b
Hyphal coils	0.1 ± 0.1% a	0.1 ± 0.1% a
Vesicles	5.4 ± 0.9% a	2.2 ± 0.4% b
Total	66.7 ± 1.6% a	54.7 ± 0.8% b

**Table 2:** Molecular identification of endophytic fungi colonizing the roots of two plants based on ITS sequences.

Isolates	Accession No.	Strain No.	Frequencies (%)	The Best BLAST Hits in UNITE			
				Organism ITS Identified	UNITE SH Code at 1.5% Threshold	Accession No.	% ITS Identity
*Podospora*			0.52				
3793	OP689613	1		*Podospora*	SH0733809.10FU	HM161911	98.14
*Humicola*			3.65				
3751	OP689614	7		*H. fuscoatra*	SH0758021.10FU	KT291422	94.58
*Cadophora*			35.42				
3621	OP689584	7		*Cadophora*	SH1011920.10FU	KT203128	100
251M	OP689591	3		*Cadophora*	SH1011920.10FU	KT269343	100
192M	OP689615	55		*Cadophora*	SH1011920.10FU	MZ674486	99.65
3232	OP689588	3		*C. luteo-olivacea*	SH1011920.10FU	OR761595	99.83
*Chaetomium*			2.6				
73P	OP689600	1		*Chaetomium*	SH0978756.10FU	MH171491	99.62
37122	OP689603	4		*Chaetomium*	SH0845223.10FU	DQ093790	94.5
*Cladosporium*			22.92				
303M	OP689590	2		*Cladosporium*	SH0962330.10FU	KX258802	100
4311	OP689594	39		*Cladosporium*	SH0962330.10FU	PP931183	99.42
75M	OP689599	3		*C. sphaerospermum*	SH0962330.10FU	OR482603	99.81
*Cyphellophora*			7.81				
3452	OP689587	15		*Cyphellophora*	SH0870058.10FU	MT453284	99.13
*Didymella*			3.125				
42Z	OP689602	4		*D. bellidis*	SH0862152.10FU	OR734621	99.6
5321	OP689605	2		*D. sinensis*	SH0862152.10FU	OP596108	100
*Dothistroma*			1.04				
245P	OP689592	2		*Dothistroma*	SH0972187.10FU	KC867932	99.39
*Endoradiciella*			1.04				
5131	OP689607	1		*E. communis*	SH0948508.10FU	PP951472	98.8
4321	OP689609	1		*E. communis*	SH0948508.10FU	PP951472	98.27
*Herpotrichiellaceae*			5.73				
5292	OP689606	11		*Herpotrichiellaceae*	SH1008212.10FU	AF373061	98.79
*Leptosphaeria*			1.56				
6411	OP689604	3		*L. sclerotioides*	SH1021671.10FU	PP348000	99.43
*Minimelanolocus*			0.52				
1411	OP689589	1		*Minimelanolocus*	SH0904736.10FU	OP626359	99.82
*Mycochaetophora*			0.52				
191P2	OP689596	1		*Mycochaetophora*	SH1011920.10FU	MT133926	99.47
*Paraphoma*			5.21				
3512	OP689585	5		*Paraphoma*	SH1022010.10FU	KT268530	99.42
196M	OP689593	5		*P. rhaphiolepidis*	SH1022010.10FU	OP482452	99.42
*Pyrenochaeta*			4.69				
43P2	OP689601	5		*Pyrenochaeta*	SH0991831.10FU	OM743888	99.03
3511	OP689586	1		*Pyrenochaeta*	SH1006324.10FU	FN394710	99.62
4411	OP689608	3		*Pyrenochaeta*	SH0991794.10FU	KU350733	100
*Pyrenopeziza*			0.52				
181M	OP689597	1		*P. atrata*	SH1011920.10FU	UDB07675204	97.6
*Rachicladosporium*			0.52				
156M	OP689598	1		*R. cboliae*	SH0962422.10FU	OQ324707	98.49
*Ragnhildiana*			0.52				
3971	OP689612	1		*R. perfoliati*	SH0972187.10FU	GU214639	99.39
*Ramichloridium*			1.04				
193P3	OP689595	2		*Ramichloridium*	SH0972027.10FU	MN065471	99.58
*Scolecobasidium*			0.52				
4312	OP689610	1		*S. constrictum*	SH0917312.10FU	LM644509	99.48
*Thysanorea*			0.52				
4061	OP689611	1		*T. asiatica*	SH0904746.10FU	KR215604	100
*Total*		192	100				

**Table 3:** Diversity indices of fungal communities colonizing the roots of *P. yunnanensis* and *C. sinica* in the abandoned tailing area of Huangmaoshan, Yunnan Province, Southwestern China. All data are presented as means ± SE (*n* = 6). Different lowercase letters in the same column indicate significant differences in the indexes between *P. yunnanensis* and *C. sinica* (student’s *t*-test, *p* < 0.05).

Plants	ACE	Chao	Shannon	Simpson
*P. yunnanensis*	108.381 ± 5.602 b	109.019 ± 4.961 b	2.679 ± 0.121 a	0.123 ± 0.016 a
*C. sinica*	54.900 ± 9.802 a	52.083 ± 10.809 a	2.304 ± 0.270 a	0.203 ± 0.077 a

**Table 4:** Siderophore activity of the representative DSE strains in culture solutions without Fe^3+^ after 5 days.

Strains	SU	As/Ar
*Cadophora luteo-olivacea* 3232	0.407	0.593
*Cyphellophora* sp. 3452	0.341	0.659
*Cladosporium* sp. 4611	0.501	0.499
*Leptosphaeria sclerotioides* 6411	0.655	0.345
*Cladosporium* sp. 151M	0.519	0.481
*Cadophora* sp. 192M	0.484	0.516

## Data Availability

The fungal ITS sequences obtained in this study were deposited in GenBank under accession numbers OP689584–OP689615. The raw sequencing data have been deposited in the NCBI Sequence Read Archive (SRA) under accession numbers SAMN31489021–SAMN31489032.

## Supplementary Materials

The following supporting information can be downloaded at: https://www.mdpi.com/article/10.3390/microorganisms12102067/s1, Figure S1: Morphological characteristics of both dark septate endophytic (DSE) and arbuscular mycorrhizal (AMF) fungi colonizing the roots of *P. yunnanensis* (A–D) and *C. sinica* (E–I) in the abandoned tailing area of Huangmaoshan, Yunnan Province, southwestern China. (A–C) Septate hyphae and DSEs in roots of *P. yunnanensis*. (D) Vesicle of AMF in roots of *P. yunnanensis*. (E) Septate hyphae of DSEs and hyphae of AMF in roots of *C. sinica*. (F) Microsclerotium of DSEs in roots of *C. sinica*. (G) Arbuscule of AMF in roots of *C. sinica*. (H) Vesicle of AMF in roots of *C. sinica*. (I) Hyphal circle of AMF in roots of *C. sinica*; Figure S2: Rarefaction curve of fungi colonizing the roots of *P. yunnanensis* and *C. sinica* in the abandoned tailing area of Huangmaoshan, Yunnan Province, Southwest China; Figure S3: Venn diagram of fungi colonizing the roots of *P. yunnanensis* (Py) and *C. sinica* (Cs) in the abandoned tailing area of Huangmaoshan, Yunnan Province, southwest China; Figure S4: CAS plate verification of the siderophore production in the 6 representative DSE strains isolated from the roots of *P. yunnanensis* and *C. sinica* in the abandoned tailing area of Huangmaoshan, Yunnan province, Southwest China; Figure S5: IAA content in the culture filtrates of the 6 representative DSE strains; Figure S6: Plant growth status of DSE-inoculated *P. yunnanensis* and their non-inoculated controls after 60-day cultivation (Bar = 7 cm).
