# Supplementary material for: Diversity and Functional Roles of Root-Associated Endophytic Fungi in Two Dominant Pioneer Trees Reclaimed from a Metal Mine Slag Heap in Southwest China
Source: Microorganisms. 2024 Oct 15;12(10):2067. doi: 10.3390/microorganisms12102067 (PMC11509953; doi:10.3390/microorganisms12102067)
Supplement: Supplementary file 1 [file microorganisms-12-02067-s001.zip › microorganisms-3232279-supplementary.pdf]

Supplementary Materials

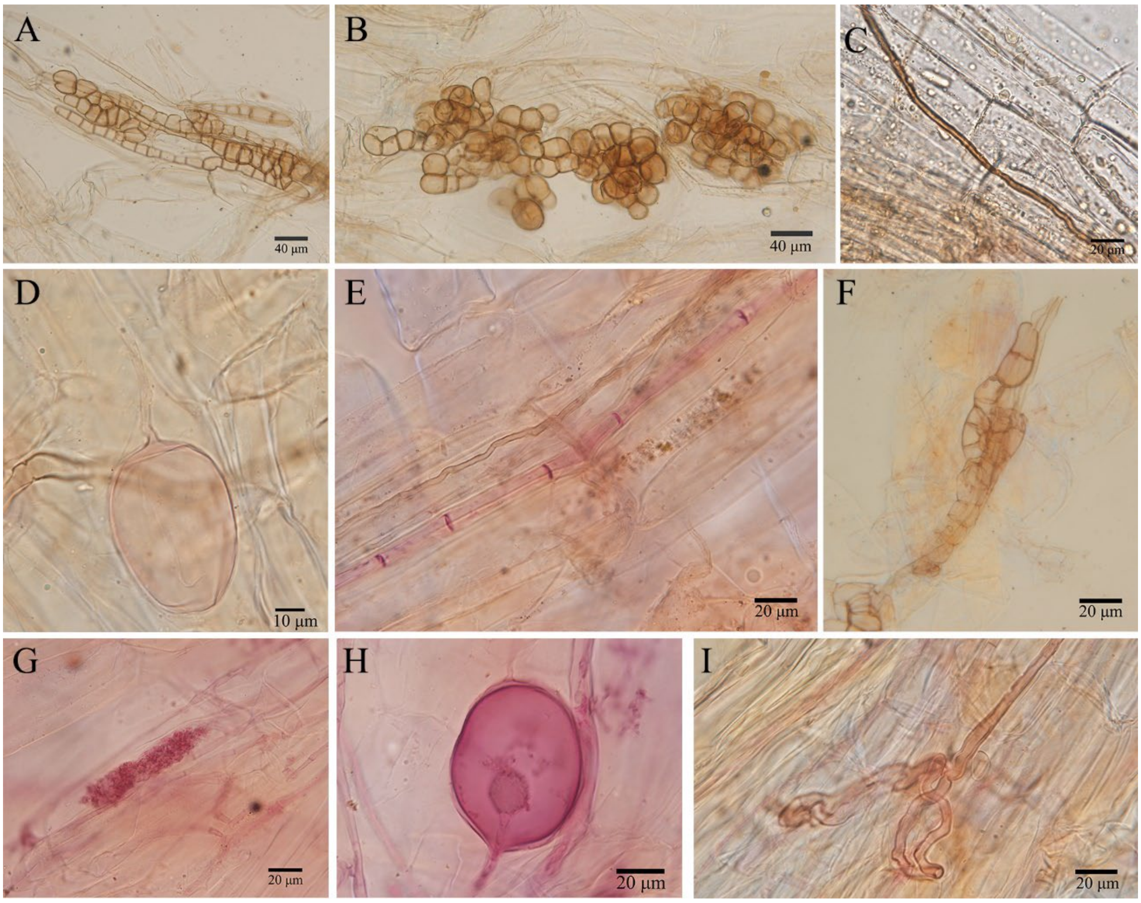

Figure S1 Morphological characteristics of both dark septate endophytic (DSE) and arbuscular mycorrhizal fungi (AMF) colonizing the roots of *P. yunnanensis* (A-D) and *C. sinica* (E-I) in abandoned tailing area of Huangmaoshan, Yunnan Province, southwestern China. A-C) Septate hyphae and DSE in roots of *P. yunnanensis*. D) Vesicle of AMF in roots of *P. yunnanensis*. E) Septate hyphae of DSE and hyphae of AMF in roots of *C. sinica*. F) Microsclerotium of DSE in roots of *C. sinica*. G) Arbuscule of AMF in roots of *C. sinica*. H) Vesicle of AMF in roots of *C. sinica*. I) Hyphal circle of AMF in roots of *C. sinica*.

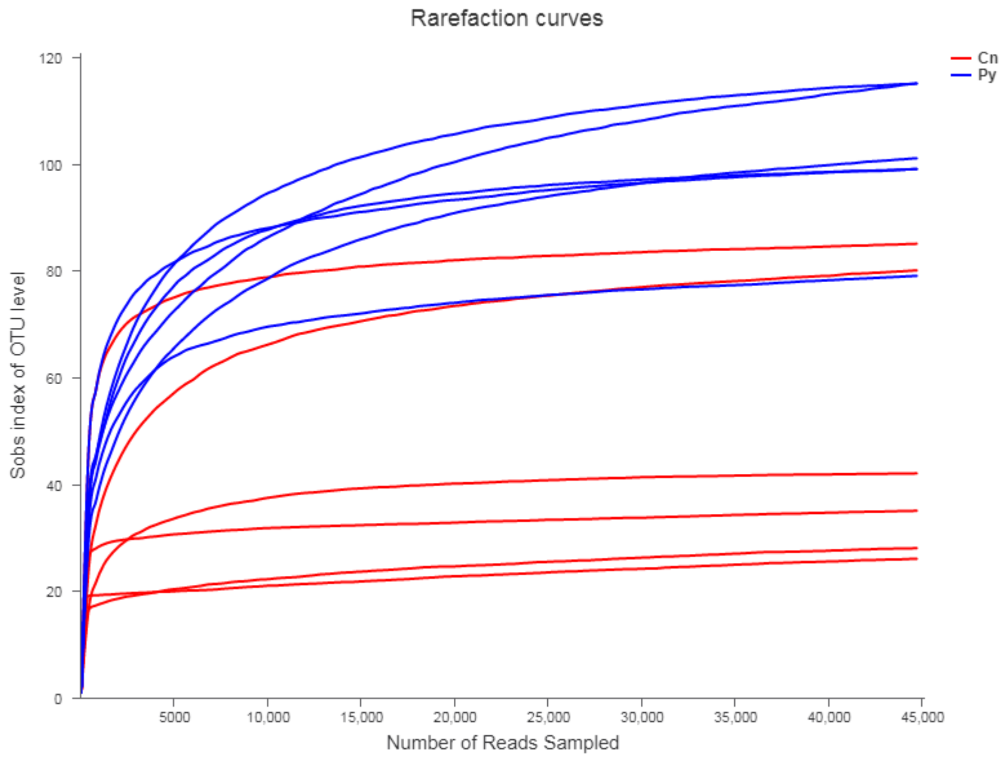

Figure S2 Rarefaction curve of fungi colonizing the roots of *P. yunnanensis* and *C. sinica* in abandoned tailing area of Huangmaoshan, Yunnan Province, Southwest China.

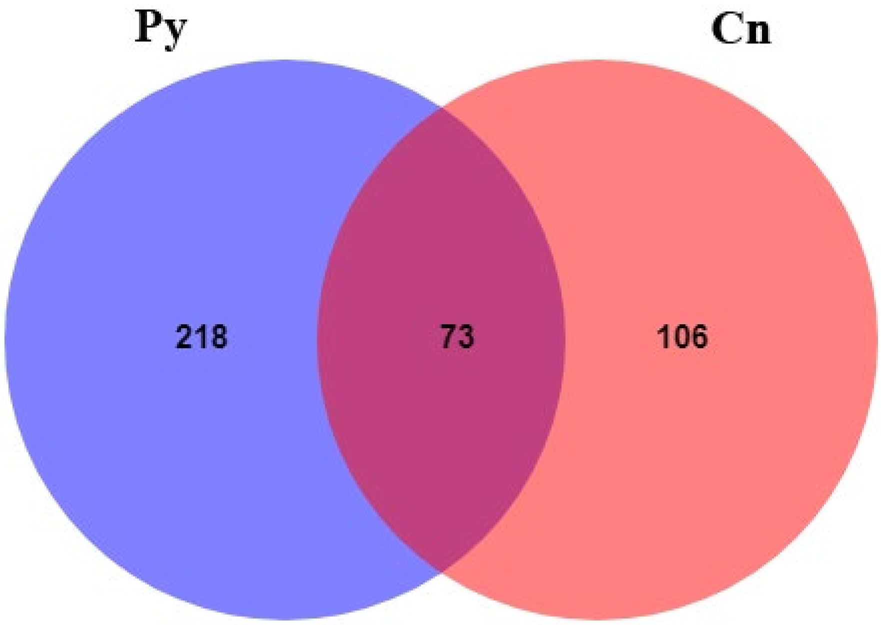

Figure S3 Venn diagram of fungi colonizing the roots of *P. yunnanensis* (*Py*) and *C. sinica* (*Cs*) in abandoned tailing area of Huangmaoshan, Yunnan Province, southwest China.

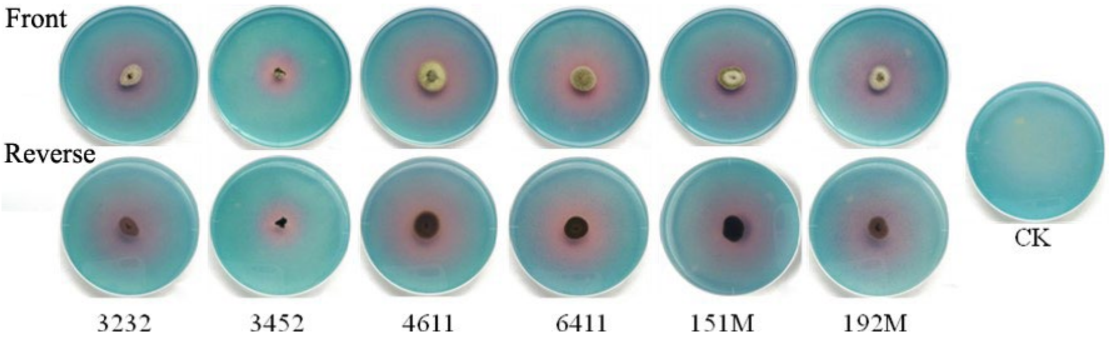

Figure S4 CAS plate verification of the siderophore production in the 6 representative DSE strains isolated from the roots of *P. yunnanensis* and *C. sinica* in abandoned tailing area of Huangmaoshan, Yunnan province, Southwest China.

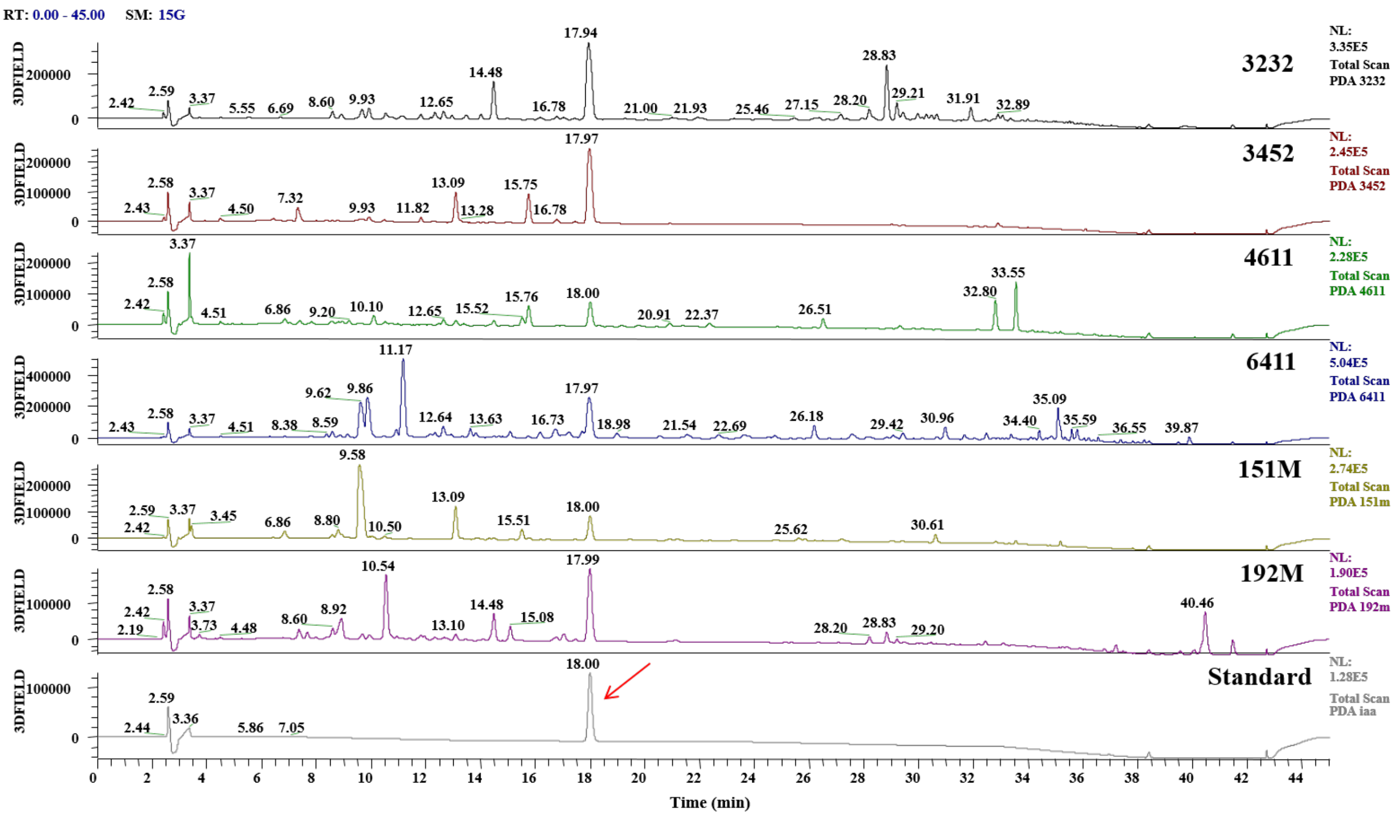

Figure S5 IAA content in the culture filtrates of the 6 representative DSE strains.

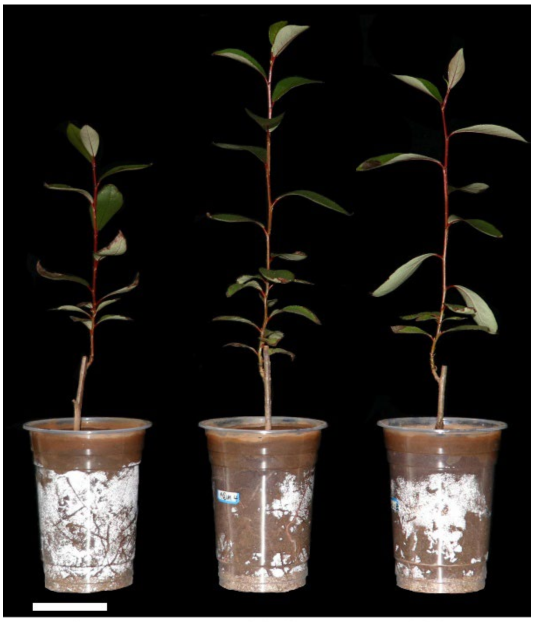

CK 3452 192M

Figure S6 Plant growth status of DSE-inoculated *P. yunnanensis* and their non-inoculated controls after 60-day cultivation (Bar = 7 cm).
